# Acid‐Responsive Dual‐Targeted Nanoparticles Encapsulated Aspirin Rescue the Immune Activation and Phenotype in Autism Spectrum Disorder

**DOI:** 10.1002/advs.202104286

**Published:** 2022-03-13

**Authors:** Xueqin He, Jiang Xie, Jing Zhang, Xiaorong Wang, Xufeng Jia, Heng Yin, Zhongqing Qiu, Zhihang Yang, Jiao Chen, Zhiliang Ji, Wenqi Yu, Meiwan Chen, Wenming Xu, Huile Gao

**Affiliations:** ^1^ Key Laboratory of Drug‐Targeting and Drug Delivery of MOE, Key Laboratory of Birth Defects and Related Diseases of Women and Children of Ministry of Education, West China Second University Hospital West China School of Pharmacy Sichuan University Chengdu 610041 China; ^2^ Department of pediatrics Chengdu Third People's Hospital Chengdu 610041 China; ^3^ State Key Laboratory of Stress Cell Biology School of Life Sciences Xiamen University Xiamen 361102 China; ^4^ State Key Laboratory of Quality Research in Chinese Medicine Institute of Chinese Medical Sciences University of Macau Macau 999078 China

**Keywords:** aspirin, autism spectrum disorder, nanoparticles, neuro‐inflammation

## Abstract

The treatment of autism spectrum disorder (ASD) is one of the most difficult challenges in neurodevelopmental diseases, because of the unclear pathogenesis research and low brain‐lesion targeting efficiency. Besides, maternal immune activation has been reported as the most mature and widely used model of ASD and aspirin‐triggered lipoxin A4 is a potent anti‐inflammatory mediator being involved in the resolution of neuroinflammation in ASD. Therefore, an aspirin encapsulated cascade drug delivery system (Asp@TMNPs) is established, which can successively target the blood–brain barrier (BBB) and microglial cells and response to the acid microenvironment in lysosome. As a result, the mitochondrial oxidative stress, DNA damage, and inflammation of microglial cells are prominently alleviated. After the treatment of Asp@TMNPs, the social interaction, stereotype behavior, and anxious condition of ASD mice are notably improved and the activation of microglial cells is inhibited. Overall, this system successively penetrates the BBB and targets microglial cells, therefore, it significantly enhances the intracephalic drug accumulation and improves anti‐neuroinflammatory efficacy of aspirin, providing a promising strategy for ASD treatment.

## Graphical abstract

An aspirin encapsulated cascade drug delivery system (Asp@TMNPs) is proposed to rescue the immune activation and phenotype in autism spectrum disorder (ASD). In this system, Asp@TMNPs successively penetrate the blood–brain barrier and target microglial cells, and then significantly enhance the intracephalic drug accumulation and improve anti‐neuroinflammatory efficacy of aspirin, providing a promising strategy for ASD treatment.

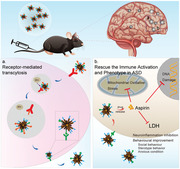

## 1 Introduction

Autism spectrum disorder (ASD) is one of the most common neurodevelopmental disorders, which is characterized by social impairment, limited interests and stereotyped behavior.^[^ ^1^ ^]^ Presently, there is about 1% prevalence of children around the world.^[^ ^2^ ^]^ The pathogenesis of ASD is still unclear, which may be affected by multiple factors, including environment and genetics.^[^ ^3^, ^4^, ^5^ ^]^ Moreover, emerging evidences have shown that the activation of microglia induced by maternal immune reaction may be a crucial factor of ASD in the offspring.^[^ ^6^, ^7^, ^8^, ^9^, ^10^, ^11^ ^]^ Our previous researches have showed that tumor necrosis factor‐*α* (TNF‐*α*), interleukin‐1 (IL‐1) and interleukin‐17 were notably increased in the serum of ASD children.^[^ ^12^ ^]^ After further association analysis, we found that the level of TNF‐*α* shows significantly positive correlation with its core symptoms, such as social interaction and self‐care, which laid a foundation for further study of relationship between inflammation and ASD.

As one of the most commonly used non‐steroidal anti‐inflammatory drugs (NSAIDs), aspirin can inhibit cyclooxygenase‐2 (COX‐2) to block the conversion of arachidonic acid to prostaglandins.^[^ ^13^ ^]^ Besides, the acetylation of COX‐2 can bind with aspirin‐triggered lipoxin A4 (ATL), which has the same anti‐inflammatory activity as natural lipoxin and is more resistant to the metabolic inactivation.^[^ ^14^ ^]^ It has been proved that ATL can induce multi‐cellular responses by interacting with lipoxin A4 receptors (ALX)^[^ ^15^, ^16^, ^17^ ^]^ and the functional ALX is expressed on multiple cells in the brain.^[^ ^18^, ^19^, ^20^, ^21^ ^]^ Among which, microglial cell is the key target and multifunctional effector of neuroinflammation,^[^ ^22^ ^]^ suggesting the potential role in the treatment of ASD. Therefore, we hypothesized the aspirin could treat ASD by inducing the LXA4 pathway activation in microglial cells.

For the aspirin‐based treatment of ASD, the selective distribution in brain especially in microglial cells, is essential for anti‐neuroinflammation with better efficiency and lower side effect. However, it needs to conquer two challenges: transportation through blood–brain barrier (BBB) and targeting the microglial cells. According to recent researches, two‐stage targeting nanoparticle is a promising solution for high and precise brain‐lesion delivery.^[^ ^23^, ^24^, ^25^, ^26^, ^27^ ^]^ Based on the receptor‐mediated transcytosis (RMT) of BBB, D‐T7 peptide (sequence: HRPYIAHC, all D‐form amino acids) can be modified on the surface of nanoparticles to enhance BBB transportation, because of its high affinity with transferrin receptors (TfR) that overexpressed on BBB.^[^ ^28^ ^]^ However, ligands cannot be easily separated from receptors after recognition and internalization,^[^ ^29^ ^]^ which would attenuate the endosome escape and transcytosis of nanoparticles, and finally lead to low brain parenchyma accumulation. Recently, our group proposed acidic cleavable ligand modification, which could greatly enhance the transcytosis of ligand modified nanoparticles through BBB.^[^ ^30^, ^31^, ^32^, ^33^ ^]^ Therefore, we employed acid‐sensitive imine linker (DAK) to conjugate D‐T7 onto nanoparticles, which would break up in the acidic environment of lysosomes (pH 4.5–6.5) to detach the nanoparticles from the “D‐T7‐TfR” complex and then enter into brain parenchyma.^[^ ^34^ ^]^ After transported into the brain parenchyma, the second‐stage ligand is needed to guide nanoparticles into microglial cells. MG1 peptide (sequence: CHHSSSAR) was obtained by three cycles of in vivo phage display screening, which had been revealed a high affinity to M1 microglial cells and could improve the treatment of therapeutic genes.^[^ ^35^ ^]^ Eventually, the acid‐responsive programmed dual‐targeted drug delivery system was established for aspirin delivery.

**Figure 1:**
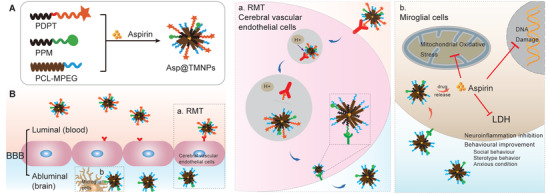
Scheme of the acid‐responsive programmed dual targeted drug delivery system (Asp@TMNPs). A) Diagram depicting the preparation of acid‐responsive programmed dual‐targeted delivery depot of Asp@TMNPs. B) Schematic illustration of Asp@TMNPs target TfR on BBB and microglial cells. Schematic diagram of process of the acid‐responsive lysosomes escaping of nanoparticles (a); Nanoparticles target microglial cells and treat ASD by targeting LDH (b).

In this study, we developed an aspirin loaded cascade targeting drug delivery system (Asp@TMNPs). The acid‐sensitive DAK was used to link polycaprolactone (PCL) and longer poly(ethylene glycol) (PEG) with D‐T7, and MG1 was modified at another shorter PCL‐PEG terminus. Then polymers were self‐assembled into nanoparticles with aspirin encapsulated (**Figure** **1**A). With the assistance of acid‐cleavable D‐T7, Asp@TMNPs targeted TfR on BBB and escaped from lysosomes’ digestion to be transported into brain parenchyma. And then nanoparticles would target to microglial cells under the guidance of MG1 and slowly release aspirin to treat ASD (Figure 1B). As a result, the mitochondrial oxidative stress, DNA damage and inflammation of microglial cells were prominently alleviated. The social interaction, stereotype behavior, and anxious condition of ASD mice were also notably improved and the activation of microglial cells was inhibited. Overall, this system successively penetrated the BBB and targeted microglial cells, thus, significantly enhanced the intracephalic aspirin accumulation and anti‐neuroinflammatory efficacy in ASD mice.

## 2 Results

### 2.1 Synthesis and Characterization of Asp@TMNPs

First, PCL was synthesized and characterized by ^1^H NMR spectrum (Figure S2, Supporting Information). Second, the acid‐sensitive thioether linker (DAK) was linked with PCL (*M*_n_ = 7164) and MAL‐PEG‐NHS to obtain PCL‐DAK‐PEG‐MAL (PDP, *M*_w_ = 11 266). D‐T7 peptide modified polymer (PCL‐DAK‐PEG‐T7, PDPT, *M*_w_ = 14 089) was then synthesized subsequently. Meanwhile, PCL‐PEG‐MAL (PP, *M*_n_ = 9278) was synthesized by the esterification of PCL and MAL‐PEG‐OH, following decoration of MG1 peptide in the PEG‐terminals of PP to form the microglia‐targeted polymer (PPM, *M*_n_ = 9509). The intermediate products were characterized by gel permeation chromatography (Figure S3, Supporting Information), respectively. Finally, PCL‐MPEG, PDPT, PPM, and aspirin were mixed proportionally to prepare nanoparticles by the emulsion/solvent evaporation method.

In vitro bEnd.3 cellular uptake was used to study the optimal modification density of D‐T7 (Figure S4, Supporting Information). The efficiency of T_5_MNPs was 1.49 and 1.35 times higher than T_1_MNPs and T_4_MNPs separately and there was no statistical difference in nanoparticles modified by more D‐T7. The results indicated that TMNPs with the molar ratio of 33.3% D‐T7 modification was the best density for brain targeting delivery, which was used in the following experiments. The hydrodynamic diameter and zeta potential of Asp@TMNPs measured by dynamic light scattering were about 62.17 nm and 8.92 mV (Table S1, Supporting Information), respectively. The drug loading capacity and encapsulation efficiency of aspirin were 19.7% and 75.2%, respectively. Transmission electronic microscopy demonstrated spherical morphology of nanoparticles with a uniform dispersity and the diameter of Asp@TMNPs was about 50 nm (Figure S5, Supporting Information).

### 2.2 Stability and Cytotoxicity of Asp@TMNPs

An excellent stability of nanoparticles is required to ensure their stable condition before administration. On the other hand, good plasma stability is the prerequisite for nanoparticles to reach the lesions after intravenous injection. After been incubated in PBS buffer for 48 h, there were no obvious changes in the hydrodynamic diameters and polydispersity index (PDI) (Figure S6, Supporting Information). However, after 24 h incubation with 10% and 50% fetal bovine serum (FBS), their hydrodynamic diameters increased ≈150–200 nm and ≈200–500 nm, respectively along with stable PDI, which may be caused by the formation of protein corona.^[^ ^36^, ^37^ ^]^ Therefore, the nanoparticles could be stored in PBS buffer for at least 48 h, but good plasma stability just maintained for 24 h which would be enough to reach the ASD lesions.

Based on the double‐side of drugs, the therapeutic effect is influenced by not only the effective drug treatment concentration in the lesion, but also acceptable toxicity. Therefore, it is essential to investigate the toxicity of Asp@TMNPs in the therapeutic concentration. According to previous studies, the intravenous dose of 40 mg kg^−1^ aspirin, equal to 90 µg mL^−1^ aspirin in cell models, was effective for mice.^[^ ^38^ ^]^ Cytotoxicity by MTT assays was measured to study the safety of Asp@TMNPs (Figure S7, Supporting Information). These results showed the cell viabilities of Asp@TMNPs were about 100%, indicating the good biosafety.

### 2.3 Evaluation of In Vitro Cellular Uptake, Acid‐Responsive Behavior and BBB Transportation of TMNPs

**Figure 2:**
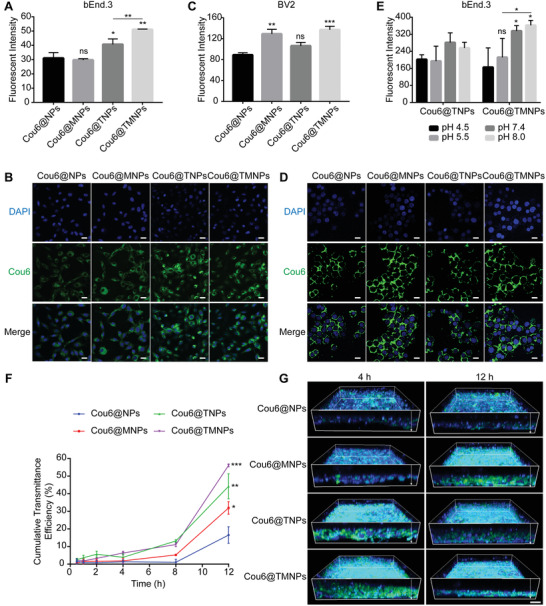
Intracellular behavior evaluation of cellular uptake, acid‐responsive behavior, and BBB transportation of TMNPs. A) Quantitative uptake of nanoparticles by bEnd.3 cells in 4 h. B) Confocal fluorescence imaging of nanoparticles taken up by bEnd.3 cells. Scale bar represents 20 µm. C) Quantitative uptake of nanoparticles by BV2 cells in 4 h. D) Confocal fluorescence imaging of nanoparticles taken up by BV2 cells. Scale bar represents 20 µm. E) Quantitative uptake of nanoparticles by bEnd.3 cells after incubated with different pH buffers for 4 h. The *p* values of Cou6@TMNPs group were comparative analysis with pH 4.5 buffer. F) Cumulative transcytosis efficiency of different formulations in the BBB model. G) 3D confocal images of bEnd.3 monolayers in the donor chamber of transwell model after the introduction of different formulations for 4 and 12 h. Scale bar represents 10 µm. All data were presented as mean ± SD (*n* = 3) and the *p* values were comparative analysis with Cou6@NPs. *p* > 0.05, 0.01 ≤ *p* < 0.05, 0.001≤ *p* <0.01, and *p* < 0.001 were remarked with ns, *, **, and ***, respectively.

A fluorescent probe, coumarin 6 (Cou6), was loaded into nanoparticles for the cellular internalization evaluation. The 8 h cumulative release ratio of Cou6 was only 0.2–4% (Figure S8A, Supporting Information), indicating the fluorescence of Cou6 could represent the nanoparticles. BBB is mainly composed by dense cerebral vascular endothelial cells, so the bEnd.3 cell is an excellent model to evaluate the penetration capacity of nanoparticles. Meanwhile, the BV2 cell model was used for the study of microglial cells’ targetability. The bEnd.3 cellular uptake efficiency of D‐T7 modified nanoparticles (TNPs) and TMNPs were 1.31 and 1.65 times higher than that of NPs, respectively with no statistical difference between MG1 modified nanoparticles (MNPs) and unmodified nanoparticles (NPs) (**Figure** **2**A,B). Above results indicated that the microvascular endothelial cell internalization of nanoparticles could be improved by D‐T7 modification. The BV2 cellular uptake efficiency of MNPs and TMNPs was 1.45 and 1.54 times higher than that of NPs separately without improvement of TNPs (Figure 2C,D), proving the MG1 modification improved microglial cell internalization of nanoparticles. Moreover, TMNPs showed the best cellular uptake behavior in both cell models, indicating the dual functions of D‐T7 and MG1 co‐modification.

Based on the RMT effect, “TfR‐nanoparticle” complex would be endocytosed into endosomes and digested by lysosomes. In view of the acid environment of lysosomes (pH ≈4.5–6.5), we designed the acid‐sensitive nanoparticles, whose lysosome escape effects had been confirmed.^[^ ^27^, ^28^ ^]^ The acid‐responsive fracture behavior of nanoparticles was verified by the bEnd.3 cellular uptake experiment (Figure 2E). Cou6‐labled nanoparticles were pre‐incubated with different pH solutions for 12 h, and bEnd.3 cellular uptake efficiency of TNPs in pH 7.4 medium and pH 8.0 medium were 1.39 and 1.27 times higher than that in pH 4.5 solutions separately. Much more significant differences were presented in TMNPs, with 2.03 and 2.19 times higher than that in pH 4.5 solutions, respectively. The down‐regulated internalization of nanoparticles demonstrated the successful breakage of D‐T7 in acid environment.

To determine the BBB transportation of TMNPs, the bEnd.3 monolayer model was established. After bEnd.3 cells were seeded, the transmembrane resistance was stable at about 170 Ω until the tenth day, and then different Cou6‐labeled nanoparticles were added into donor chamber of transwells to illustrate their transmembrane efficiency by 3D confocal imaging (Figure 2G). The longitudinal (*Z*‐axis) displayed that the TMNPs penetrated faster and deeper than control nanoparticles. The cumulative transmittance efficiency of nanoparticles showed that there was no obvious difference of each group incubated for 8 h, but TMNPs increased rapidly and revealed significant difference between 8 and 12 h (Figure 2F). The 12 h cumulative transmittance efficiency of TMNPs, TNPs, and MNPs were 3.39, 2.67, and 1.93 times higher than that of NPs, respectively, indicating the significant BBB transcytosis enhancement of D‐T7 modification and the synergistic effect of MG1. These results demonstrated the superiority of acid sensitive D‐T7 peptide modification in helping the nanoparticles transporting through BBB.

### 2.4 Evaluation of In Vitro Cellular Mitochondrial Oxidative Stress, DNA Damage, and Inflammation Cytokine Expressions with of Asp@TMNPs and Identifying Its Potential Target

**Figure 3:**
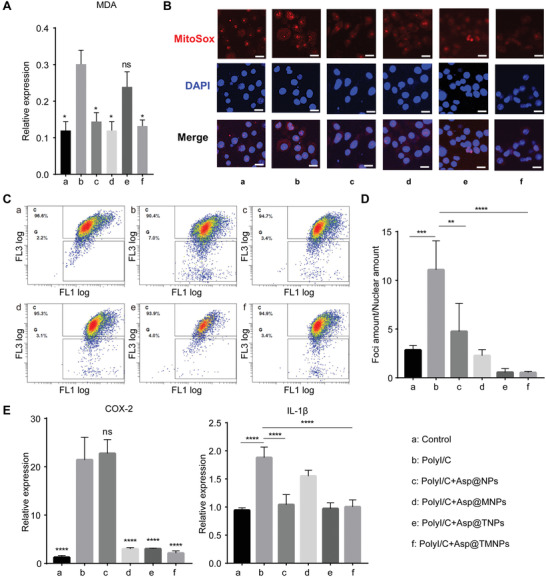
Evaluation of in vitro cellular MDA levels, mitochondrial oxidative stress, DNA damage and cytokine expressions with Asp@TMNPs. A) The MDA concentration in different groups. Data were presented as mean ± SD (*n* = 3). B) The MitoSox staining after different groups of treatment, red showed MitoSox staining. Scale bar represents 20 µm. C) Flow cytometry results of JC‐1 staining in PloyI/C treated group, as well as different formulations of aspirin. D) The *γ*H2AX foci numbers in different treatment groups as shown in the bar chart. Data were presented as mean ± SD (*n* = 3). E) The real time PCR results showed the expression of inflammation cytokines of COX‐2 and IL‐1*β* after Asp@TMNPs treatment. Data were presented as mean ± SD (*n* = 3). In this figure, a: control group (Control), b: PolyI/C transfection group (PolyI/C), c: PolyI/C+Asp@NPs, d: PolyI/C+Asp@MNPs, e: PolyI/C+Asp@TNPs, and f: PolyI/C+Asp@TMNPs. All *p* values were comparative analysis with PolyI/C, and *p* > 0.05, 0.01≤ *p* <0.05, 0.001 ≤ *p*<0.01, *p* < 0.001, and *p* < 0.0001 were remarked with ns, *, **, *** and ****, respectively.

To analysis whether polyinosinic acid‐polycytidylic acid (PolyI/C transfection could induce excessive oxidative stress, we measured the BV2 cellular oxidative parameters, including MDA and ROS. The concentration of MDA was significantly increased around three times after PolyI/C incubation, while the levels showed dose‐dependent decrease after Asp@TMNPs treatment (**Figure** **3**A). To further study the changes of mitochondrial ROS, we used MitoSox red probe to determine the ROS level and the immunostaining result showed that while PolyI/C treatment induced MitoSox levels, the Mitosox level is significantly reduced after intervention of aspirin (Figure 3B). We then used JC‐1 probe to determine whether PolyI/C caused JC‐1 mono‐aggregate (green fluorescence), and the rescue effect of aspirin. The flow cytometry result showed that aspirin could effectively reduce the mono‐aggregate formation, though the different formula show similar effects (Figure 3C).

One of the most significant effects of oxidative stress is DNA damage and DNA double‐strand break (DSB) is thought as the most severe form of DNA damages. It has been widely accepted that 139 site phosphorylation as marked as *γ*H2AX foci could be used to evaluate the DNA damage level. We then used immunostaining to examine the effect of DNA damage in microglia cell after PolyI/C treatment. The result showed that the number of foci was significantly increased after Poly I/C treatment, while the increased foci number was inhibited significantly (Figure 3D; Figure S9, Supporting Information). To further determine whether the increased foci could cause increased inflammation response, we checked the expression of inflammation related cytokines, including IL‐1*β* and COX‐2. qPCR result showed that PolyI/C treatment induced COX‐2 and IL‐1*β* gene expression. Furthermore, the levels of inflammation cytokines including COX‐2 and IL‐1*β* were significantly reduced after Asp@TMNPs treatment (Figure 3E).

**Figure 4:**
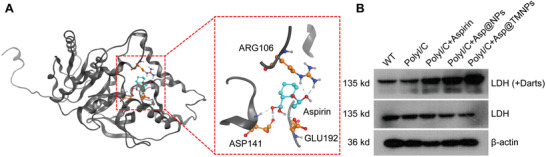
The potential target of aspirin in microglial cells. A) The molecular docking result of the aspirin with its potential target, LDH. B) The Darts result of the different drugs treatment in microglia cells. The first lane is the Darts result. BV2 cells lysates were incubated with, 0.12 mg mL^−1^ Pronase E in the medium and the LDH level was detected. The second lane is the western blot lane of LDH, and *β*‐actin was used as loading control.

Since it has been shown that Asp@TMNPs can inhibit inflammation and oxidative stress, it could provide more mechanism insight if the potential target of aspirin could be identified in microglia cell. We then used PharmMapper, a widely used on‐line database to predict the potential target of aspirin in microglial cell. The result showed that l‐Lactate dehydrogenase I (LDH) as the Rank 1 candidate target of aspirin, which was demonstrated by molecular docking (**Figure** **4**A). We then used drug affinity responsive target stability (Darts), an established method to confirm the direct binding of LDH with aspirin,^[^ ^39^ ^]^ and our Darts result showed that LDH band was protected from Protase E digestion by the presence of aspirin in microglial cells (Figure 4B). And Western blot result showed that compared with free aspirin, Asp@TMNPs treatment leads to more significantly reduced expression of LDH in microglial cells, indicating that Asp@TMNPs can inhibit mitochondrial oxidative stress and immune activation through targeting LDH, at least in vitro.

### 2.5 In Vivo Distribution of TMNPs

**Figure 5:**
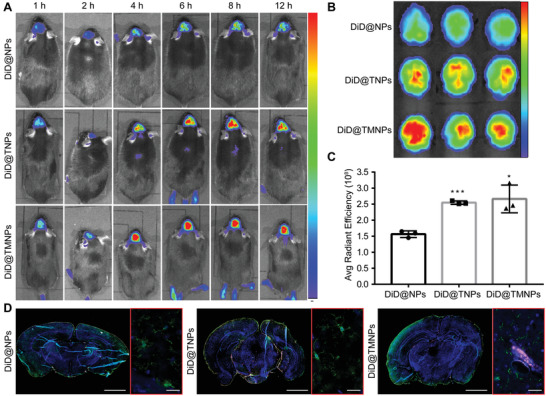
In vivo distribution showing different distribution of nanoparticles in brain slice. A) Living imaging depicting the in vivo distribution of different formulations at different time. B) Ex vivo images of brain in different groups after 12 h administration. C) The semiquantitative fluorescence intensity of brains. Data were presented as mean ± SD (*n* = 3). 0.01 ≤ *p* < 0.05 and *p* < 0.001 were remarked with * and ***, respectively. D) Representative confocal fluorescence images of brains showing the accumulation of different nanoparticles. Scale bar represents 50 µm.

To explore the in vivo BBB transportation and brain accumulating capacity of nanoparticles in ASD, DiD was loaded in nanoparticles physically, and the 24 h cumulative release efficiency of DiD was only 0.02–0.1% (Figure S7B, Supporting Information), indicating the fluorescence of DiD could represent the nanoparticles. The biodistribution of DiD‐labeled nanoparticles in ASD mice model were monitored by living imaging (**Figure** **5**A). The brain fluorescent intensity of mice administrated with DiD@TMNPs or DiD@TNPs were continuously enhanced and increased to the top between ≈8–12 h, while there were no obvious changes in NPs group. At 12 h post injection, mice were sacrificed and ex vivo images of main organs were obtained (Figure 5B; Figure S10A, Supporting Information). Although high level of nanoparticles was distributed in livers, the brain fluorescence intensity of TNPs and TMNPs was much stronger than NPs group, which was 1.62 and 1.70 times higher according to semi‐quantification (Figure 5C). The results demonstrated that D‐T7 modification endowed faster BBB transportation and more brain accumulation of nanoparticles in ASD mice. Ex vivo peripheral organ images of each group showed the similar distribution (Figure S10B, Supporting Information).

Brains were also sliced to observe the accumulation of nanoparticles and immunofluorescence colocation with microglial cells by confocal imaging (Figure 5D). The brain fluorescence intensity of TMNPs was much stronger than others, and the obvious microglia immunofluorescence colocation image was observed in MG1 modified nanoparticles. Moreover, there were no obvious differences in peripheral organs distribution (Figure S10C, Supporting Information). These results indicated that D‐T7 could effectively improve brain targeting and MG1 could further promote the microglia targeting delivery.

### 2.6 The Social Behavior Parameters Measured by Three‐Chamber Test

**Figure 6:**
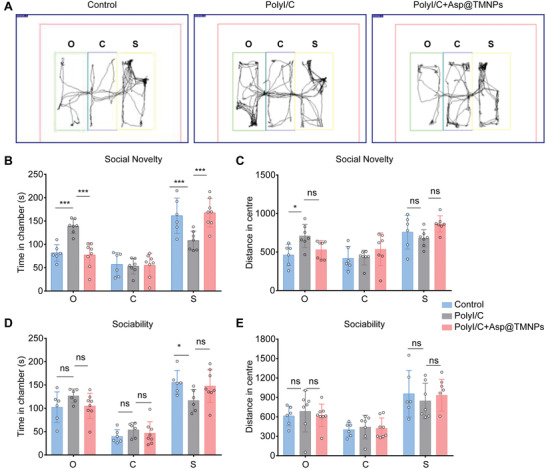
Three‐chamber test of ASD mice administrated Asp@TMNPs. A) The representative traces of ASD mice in three chamber social test. B) In social study, the total spend time in stranger chamber was compared in different mice groups. C) The distance in center was also compared in different groups, including Control, PolyI/C and Asp@TMNPs group. D) In social study, the time in chamber was compared in different groups. E) In social test, the distance spent in strange chamber and familiar chamber was compared in different groups. O, C and S presents object, central and strange chamber, respectively. All data were presented as mean ± SD (*n* = 6). *p* > 0.05, 0.01 ≤ *p* < 0.05, and *p* < 0.001 were remarked with ns, * and ***, respectively.

Based on the in vitro microglial cell result, we further aim to determine whether PolyI/C can induce the neurobehavioral abnormality and whether aspirin can rescue the result in vivo. We used three‐chamber social test to determine the social‐behavior abnormality and our result showed that PolyI/C treated mouse had longer time to familiar mouse, while the time with strange mouse was reduced (**Figure** **6**A,B). Furthermore, mouse treated with Asp@TMNPs had longer time to stay with strange mouse, while significantly reduced time with the central objects. On the other hand, there was no significant difference in the social contact time among the groups (Figure 6C,D) as well as the distance in center (Figure 6E). Together, the result showed that Asp@TMNPs could effectively rescue some aspects of the social deficiency caused by PolyI/C treatment during pregnancy.

### 2.7 Stereotype Behavior and Anxious Condition Measured by Elevated Plus‐Maze, Open‐Field and Self‐Grooming Test

**Figure 7:**
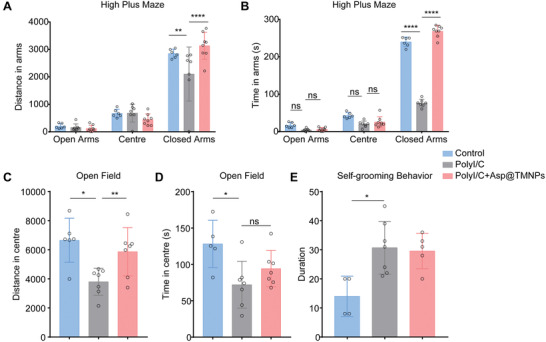
Stereotype behavior and anxious condition Measured by elevated plus‐maze, open‐field and self‐grooming test. A,B) The distance and the time spent in the closed arm were compared between different groups. C,D) Open‐field test shows the traveled distance and the time in the center of animals in different groups. E) The time of grooming behaviors were compared with different groups. All data were presented as mean ± SD (*n* = 6). *p* > 0.05, 0.01 ≤ *p* < 0.05, 0.001 ≤ *p* < 0.01, and *p* < 0.0001 were remarked with ns, *, ** and ****, respectively.

To further determine whether infection of pregnancy may lead to neurobehavior abnormality related to ASD, we further examine whether pregnancy infection may lead to anxious condition. The result showed that in the elevated plus‐maze test, offspring of female mice injected with PolyI/C during pregnancy spent less time in the closed arm than that of progenies from control pregnant animals treated with saline (control group) (**Figure** **7**A,B). However, both time and traveled distance in the closed arm were increased in offspring of dams treated with Asp@TMNPs after PolyI/C injection.

Results from the open‐field test showed decreased traveled distance and time in the center of young animals from PolyI/C group (Figure 7C,D). This reduction was reversed by Asp@TMNPs treatment, but without significant differences in time spent in the center. Offspring of PolyI/C group showed dramatically increased grooming behaviors. But Asp@TMNPs treatment failed to modulate the grooming behaviors (Figure 7E).

### 2.8 In Vivo Morphology Changes of Microglial Cells

**Figure 8:**
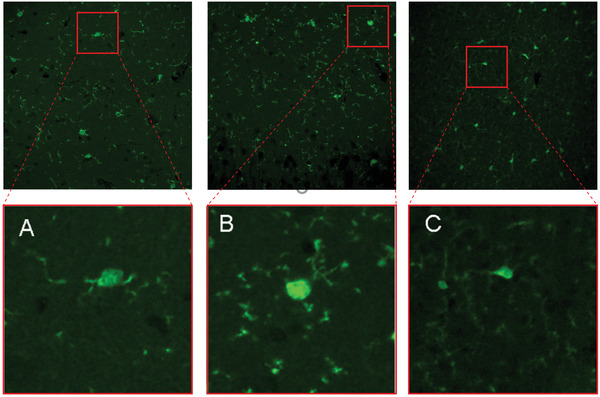
Immunostaining of CD11b marker to show the microglia activation in in vivo condition. A) The microglia cell immunostaining of CD11b marker was shown in the slide. B) PolyI/C induced increased staining in CD11b. C) Asp@TMNPs inhibited CD11b staining compared with PolyI/C treatment group. The down‐panel figure is the enlarged picture of up‐panel.

One of major phenotype in pregnancy infection is microglia cell activation. To evaluate the microglia cell activation, brain section was prepared and immunofluorescence staining of microglia cell marker, CD11b was used to detect the microglia activation. The result showed that compared with control group, PolyI/C group had significantly increased staining of CD11b, indicating activated microglia cells, while the activation was obviously reduced after in vivo treatment of Asp@TMNPs (**Figure** **8**).

## 3 Discussion and Conclusion

Neurodevelopmental diseases represented by ASD are a huge medical burden for the affected family and the whole society. Although the etiology remains elusive, infection and inflammation during pregnancy are considered to be key etiological factors of ASD.^[^ ^40^ ^]^ It has been proved that PolyI/C treatment in animal model could lead to elevated local cytokines release, including TNF and IL‐1*β*, thus indicating that Poly I/C treatment during pregnancy may lead to brain inflammation in offspring. Recent studies also showed that Th‐1 related cytokines have combinational effect on the synaptical connectivity of human neuron cell lines.^[^ ^41^ ^]^ Therefore, the mouse model elicited by PolyI/C application could recapitulate the key symptoms of autism and be used to examine the efficacy of the candidate remedies, especially for maternal immune activation (MIA) related autism. Based on current research results, we have found that a novel designed acid‐responsive programmed dual‐targeted nanoparticles encapsulated aspirin could effectively rescue the immune activation and phenotype in the immune activated ASD mouse model. In agreement with previous study, the current study indicated that PolyI/C lead to microglia activation, social deficiency, and elevated cytokines release. Compared with aspirin alone, the Asp@TMNPs showed excellent effects on the rescue of key symptoms in the ASD mouse model.

The key finding of the current studies is that the nano‐carrier provides a significant internalization of aspirin. The highly restrictive BBB is the biggest limitation of the drugs to treat central nervous system (CNS) disorders in achieving efficient transportation and reaching the lesion. With the rapid development of nanotechnology, it is expected that newly developed drug delivery systems would have a significant improvement in the diagnosis and therapy of CNS disorders.^[^ ^42^ ^]^ RMT is the most common mechanism used by ligands‐modified nanocarriers to traverse the BBB.^[^ ^43^ ^]^ In the current study, we prepared D‐T7 decorated, 60 nm nanoparticles with an acid‐cleavable diamino ketal linker between the D‐T7 and nanoparticles core. The high‐affinity of D‐T7 with TfR could efficiently mediate targeting delivery of nanoparticles to brain lesion site. The acid‐cleavable linker may be beneficial for facilitating the transcytosis of NPs across BBB because it can realize the dissociation of nanoparticles from “D‐T7‐TfR” complex. Meanwhile, entering the brain parenchyma did not mean the therapeutic effect of drug, and there is a need to endow nanoparticles with the following target delivery to the lesion. In our study, we further decorated MG1, an 8‐amino acid peptide, onto the nanoparticles before modification with D‐T7. The developed acid‐cleavable D‐T7 and MG1 co‐modified drug delivery system (TMNPs) exhibited an excellent biological performance of enhanced BBB transcytosis and precise microglial targeting delivery, as well as improved rescue effect of the key symptom in ASD model.

In vitro bEnd.3 cellular uptake showed that the internalization of acid‐sensitive TMNPs and TNPs were much higher than NPs and MNPs, suggesting that D‐T7‐coating could facilitate the internalization of nanoparticles into brain microvascular endothelial cells. Besides, in vitro BV2 cellular uptake proved that the internalization of TMNPs and MNPs were much higher than NPs and TNPs, indicating that MG1‐coating could improve the internalization of nanoparticles into microglial cells. In vitro bEnd.3 monolayers transcytosis demonstrated that TMNPs in basal well was higher than others, suggesting the acid‐cleavable and dual‐target modification property could promote the nanoparticles transporting across BBB. In vivo fluorescence imaging showed the cumulative fluorescence signal of D‐T7‐coating nanoparticles in brains was higher than PEGylated nanoparticles, while the introduction of cleavable D‐T7 greatly improved nanoparticles’ accumulation in the brain. The colocalization with microglia by immunofluorescence showed the highest affinity of TMNPs in different nanoparticles, indicating the improved microglia tendency of MG1 modified nanoparticles.

Aspirin has been widely used for the treatment or prevention of thromboembolic and neurological disease, such as ischemic cerebrovascular shock.^[^ ^44^ ^]^ However, it remains controversial whether it could be used for mental disease, such as depression. Furthermore, adverse effects presented by bleeding also limit its usage. Herein, using an intelligent system, we have found that compared with aspirin alone or non‐targeting control, the Asp@TMNPs can improve the efficacy for inhibiting microglial inflammation, cytokine release. Furthermore, to further investigate the potential targets, we used PharmMapper, an on‐line database to predict the potential target. Using Darts, our result showed that LDH was the potential target of aspirin in microglial cells. Previous study has shown that reduced LDH expression could repress inflammation in microglial cells.^[^ ^45^ ^]^ Therefore, the current study supports the notion that aspirin can inhibit the inflammation in microglial cell by targeting LDH. After loading aspirin into nanoparticles, Asp@TMNPs can greatly improve the microglial targeting efficiency in the animal model, thus it represents a novel strategy for the smart design to treat ASD. The design with aspirin may inspire the novel treatment of the other neurological diseases, such as spinal cord repair, depression, and other mental diseases.^[^ ^46^, ^47^, ^48^, ^49^ ^]^

In summary, we designed an acid‐sensitive and dual‐targeted drug delivery depot (Asp@TMNPs), which could progressively target BBB and microglial cells for improving the treatment of aspirin in ASD. First, cellular uptake efficiency validated that D‐T7 peptide could specifically recognize the TfR on the brain microvascular endothelial cells and increase the transportation of nanoparticles through RMT. Besides, MG1 peptide could characteristically distinguish microglial cells and improve the internalization of nanoparticles. Second, in vitro BBB model transcytosis and in vivo fluorescence imaging investigation demonstrated that dual‐targets modified nanoparticles could quickly and effectively pass through BBB and target to microglial cells. Finally, given the long history of safety and anti‐inflammation effect of aspirin, Asp@TMNPs represented a promising avenue for future development of the target therapy of the brain related disease, inducing autism and depression.

## 4 Experimental Section

### Materials, Cells, and Animals

MPEG‐PCL (*M*_n_ = 3400:15 000) was provided by Prof. Jianyuan Hao (University of Electronic Science and Technology of China). MG1 (sequence: CHHSSSAR) was synthesized by Sangon Biotech Co. Ltd. (Shanghai, China). The D‐T7 peptide with a cysteine on C‐terminal (D‐T7‐cys) (sequence: HRPYIAHC, all D‐form amino acids) was synthesized by Phtdpeptides Co., Ltd. (Zhengzhou, China). Maleimide PEG succinimidyl carboxymethyl ester (MAL‐PEG‐SCM, *M*_n_ = 5000) and Maleimide PEG hydroxyl (MAL‐PEG‐OH, *M*_n_ = 3500) were purchased from Jenkem Technology Co. Ltd. (Beijing, China). The Iba‐1 antibody and FITC goat anti‐rabbit IgG (H+L) were purchased from Abcam (USA). The goat serum was purchased from Solarbio (China). The bEnd.3 and BV2 cell lines were obtained from the Chinese Academy of Sciences Cell Bank (Shanghai, China). Dulbecco's Modified Eagle's Medium (DMEM), trypsin‐EDTA solutions, and FBS were purchased from Gibco (USA). The anti‐GAPDH (1:5000, EM1101, Huabio), HRP conjugated goat anti‐mouse IgG (1:5000, ZB‐2305, Zsbio) and HRP‐conjugated goat anti‐rabbit IgG (1:5000, ZB‐2301, Zsbio). The anti‐*γ*H2AX (1:400, ab26350, Abcam), goat anti‐mouse antibody, Alexa Fluor594 (1:1000, A11005, Invitrogen) and goat anti‐rabbit antibody Alexa Fluor 594 (1:1000, A11012, Invitrogen). All of the other chemicals were analytical or reagent grade.

### Quantified Fluorescence Intensity by Flow Cytometry

The bEnd.3 and BV2 cells were seeded at the density of 5 × 10^4^ cells per well in 12‐well plates and cultured at 37 °C for 36 h, respectively. The Cou6‐labled nanoparticles (200 ng mL^−1^ Cou6) and cells were incubated together for 2 or 4 h at 37 °C. Then cells were washed three times with PBS buffer. The single cell suspension was prepared and quantified by flow cytometry (BD FACS Celesta, USA).

### Qualitative Fluorescence Intensity by Confocal Microscopy

The same cell treated procedure was operated in 6‐well plates as the quantified analysis. After the different treatment, slides adhered cells were fixed with 4% paraformaldehyde for 30 min and then stained with 0.5 µg mL^−1^ DAPI for 5 min. Fluorescence intensity was observed with laser scanning confocal microscopy (Eclipse Ti, Nikon, Japan).

### In Vitro BBB Model Transcytosis

The nanoparticles encapsulated with Cou6 were added into bEnd.3 monolayer coated transwells. At 0.5, 1, 2, 4, 8, and 12 h after injection, the fluorescence intensity of liquid in the lower chamber was measured separately by a Fluorescence Spectrophotometer (Labsolutiongs RF, Shimadzu, Japan). After 4 and 12 h, bEnd.3 monolayers in the plate were processed and imaged by the confocal microscope.

### Drug Target Screening and Target Validation with Darts

To confirm the drug target of the aspirin, PharmMapper, a widely used on‐line database to predict the potential target of aspirin was chosen. To further confirm whether aspirin can bind with LDH, Darts were used. In brief, cell lysates were diluted with same cell volume and then proteolysis was performed with Pronease E (0.12 mg mL^−1^) for 5  min and stopped with 99 °C for 10 min.

### Predict the 3D Structure of LDH

The 3D structure model of LDH protein was built on the basis of the crystal structure of LDH from Homo sapiens (PDB entry: 5W8L) by using SWISS‐MODEL server.^[^ ^50^, ^51^, ^52^, ^53^, ^54^ ^]^ Then the predicted structure model was optimized with 50 ns by using GROMACS.^[^ ^55^ ^]^

### Molecular Docking

AutoDock 4.2 software was used to perform molecular docking of aspirin with LDH.^[^ ^56^ ^]^ The lowest energy docking model from the resulting 100 docking models was selected to perform molecular dynamics simulation with 50 ns by GROMACS.

### In Vivo Imaging

The model mice were vein‐intravenously injected at the dose of 0.5 mg kg^−1^ DiD (50 mg kg^−1^ TMNPs) with nanoparticles labeled by DiD (DiD@TMNPs and control nanoparticles). After injection for 1, 2, 4, 6, 8, and 12 h, the ASD mice were imaged using the Lumina III Imaging System (PerkinElmer, USA). At 12 h, the mice were sacrificed and their organs (heart, liver, spleen, lung, kidney, and brain) were separated, which were captured as above. All tissues were totally soaked into 4% paraformaldehyde for 24 h, then dehydrated with 10% and 30% sucrose solution for 24 h separately and embedded in Tissue‐Tek O.C.T compound (Sakura Finetek, USA). Then they were sectioned at 10 µm with the freezing microtome (Leica CM1950, Germany). Brain slides were operated by immunofluorescence of anti‐CD11b (bs‐1014R‐AF488, Bioss) antibody and others were stained with DAPI. The conditions of co‐localization were observed using a confocal microscope. All animals were maintained under SPF grade feeding conditions and experiments were approved by the Animal Experimentation Ethics Committee of Sichuan University (KS2020420).

### Flow Cytometry

For JC‐1 (C2006, Beyotime) staining, 1 × 10^6^ cells were resuspended in 0.5 mL cell culture medium. JC‐1 working solution (0.5 mL) was added and then mixed before incubating for 20 min at 37 °C. During incubation, 1 mL JC‐1 buffer solution (1×) was prepared with H_2_O (4 mL) and stock solution (5×), and then the solution was put on the ice solution. After 37 °C incubation, the cells were centrifuged with 600 g ≈3–4 min at 4 °C, then after the centrifuge, supernatant was discarded, and the cells were resuspended to use cytometry to measure the JC‐1 positive cells (Beckman).

### Behavioral Test

All the behavior tests were carried out between PND42‐56.

### The Three‐Chamber Social Test

The three‐chamber device was used to test the social communication ability of different groups of mice. The apparatus consisted of three Plexiglas chambers (40 cm × 20 cm × 20 cm) with the side chambers each connected to the middle chamber by a corridor (10 cm × 10 cm × 15 cm). One side of the compartment was set up with unfamiliar mouse of the same sex and age which had no previous contact, while the other side a toy. At the beginning of the test, the mouse was placed into the middle chamber and allowed the exploration of the three chambers for 5 min. Then an unfamiliar mouse, locked in a small cage, was placed in one of the side chamber and a familiar mouse was placed in the other side chamber. The testing mouse was allowed to freely explore the apparatus and interact with them for 10 min. The activity of mouse in the set was recorded, and the time of communication with unfamiliar mouse and object was counted. Social behaviors were analyzed using a social behavioral analysis system (BW‐Social LAB, Shanghai Biowill Co. Ltd).All behavioral experiments were carried out during the dark period of the light cycle under dim red illumination.

### The Open‐Field and Self‐Grooming Test

The open‐field experiment device (40 cm × 40 cm) was used to detect the mice's anxious and stereotypical self‐grooming behavior. Before the test, the mice were placed in the device for 5 min, and then mice behavior was recorded for 10 min, and their self‐grooming behaviors were counted. Social behaviors were analyzed using a social behavioral analysis system (BW‐Social LAB, Shanghai Biowill Co. Ltd.). Grooming were recorded by two trained observers who were blinded to the experimental conditions and grooming was defined as self‐licking of the body and/or self‐rubbing of the face or fur with the front paws.

### The Elevated Plus Maze Test

The mice anxious‐like behaviors were detected by using elevated cross maze experimental device. This device has two open arms and two closed arms (two arms with 20 cm high walls on both sides), a length of 50 cm on each side. There was a connected platform (10 cm × 10 cm) in the middle. The mice were placed on the intermediate platform of the device, and the cameras were used to record for 10 min, including the time and times of their entering the open and closed arms.

### Immunofluorescence

Frozen tissue of brain slices were washed and blocked after the animals were sacrificed and processed. Then the slices were incubated with primary anti CD11b antibody overnight at 4 °C, and then the slices were further stained with Alexa 488‐conjugated secondary antibodies (1:1000, Life Technology) at room temperature for 2 h. Then after staining with DAPI solution, the sections were sealed by anti‐fade mounting medium. Images were captured by a confocal microscope (F1000, Olympus).

### Statistical Analysis

The paired Student's two‐tailed two‐sample *t*‐test for the hypothesis of equal variances was used in the comparative analysis of each group. Sample size (*n*) for each statistical analysis was presented in corresponding legend. *p* > 0.05, 0.01 ≤ *p* < 0.05, 0.001 ≤ *p* < 0.01, *p* < 0.001 and *p* < 0.0001 were considered a statistically significant difference and remarked with ns, *, **, *** and ****, respectively.

## Conflict of Interest

The authors declare no conflict of interest.

## Supporting information

Supporting Information

## Data Availability

Research data are not shared.
