# Supplementary material for: Acid‐Responsive Dual‐Targeted Nanoparticles Encapsulated Aspirin Rescue the Immune Activation and Phenotype in Autism Spectrum Disorder
Source: Adv Sci (Weinh). 2022 Mar 13;9(14):2104286. doi: 10.1002/advs.202104286 (PMC9108608; doi:10.1002/advs.202104286)
Supplement: Supplementary file 1 — Supporting Information [file ADVS-9-2104286-s001.pdf]

Supporting Information

Acid-responsive Dual-targeted Nanoparticles Encapsulated Aspirin Rescue the Immune Activation and Phenotype in Autism Spectrum Disorder

Xueqin He†, Jiang Xie† , Jing Zhang, Xiaorong Wang, Xufeng Jia, Heng Yin, Zhongqing Qiu, Zhihang Yang, Jiao Chen, Zhiliang Ji, Wenqi Yu, Meiwan Chen, Wenming Xu and Huile Gao**

1. Supporting methods

Synthesis of PCL: Polycaprolactone (PCL) was synthesized according to a previous study (Figure S1). At first, calcium hydride (457.8 mg) and ε-caprolactone (15 mL) were added into a 100 mL round bottom flask and distilled under reduced pressure to obtain dry ε-caprolactone. And then dry ε-caprolactone (1.1082 mL) and decanoic acid (129.2 μL) were added into a 50 mL round bottom flask. After stirring for 7 h at 230℃ under the protection of argon, the reaction products were cooled down to room temperature and changed from a pale yellow liquid to a white waxy solid. At last, the reaction solid was firstly dissolved into tetrahydrofuran (15 mL) and precipitated to methanol by dropwise addition, which would be repeated for 3 times. The precipitated powders were then collected and recrystallized for 3 times to yield the white solids.

Synthesis of PCL-PEG-MAL (PP): PCL (15 mg), MAL-PEG$_{3500}$-OH (20 mg), 4-dimethylaminopyridine (1 mg) and 1-(3-dimethylaminopropyl)-3-ethylcarbodiimide (EDC, 19.2 mg) were dissolved into dry dichloromethane (DCM, 5 mL). After kept stirring for 24 h at room temperature, the reaction mixture was evaporated under vacuum to completely remove the organic solvent. Then the creamy yellow reaction product was dissolved into dimethyl sulfoxide (DMSO, 5 mL) and was dialysis for 24 h in DMSO and water. PP was obtained by freeze-drying.

Synthesis of microglia-targeted PCL-PEG-MG1 (PPM): The MG1 peptide (1 mg) and PP (10 mg) were dissolved into 3 mL acetone, and then TEA (15 μL) were added to maintain the

alkaline environment. After continued stirring for 12 h at room temperature, the mixed solvent was evaporated under vacuum to completely remove the organic solvent, and then dissolved into DMSO (2 mL) again. The mixture was collected after dialysis in DMSO and deionized water for 24 h, respectively using dialysis tube (3500 Da). After removed unreacted ingredients, the solution was lyophilized and the final product was obtained.

Synthesis of acid-sensitive cleavable PCL-DAK-PEG-MAL (PDP): PCL (80 mg), EDC (10.4 mL) and N-hydroxysuccinimide (12.4 mg) were dissolved into dry DCM (10 mL). After kept stirring for 2 h at room temperature, DAK (8.8 mg) and triethylamine (TEA, 5.6 μL) were added into the reaction mixture to continue string for 12 h. And then MAL-PEG5000-SCM (133.6 mg) was introduced to continue reacting for 12 h. Finally, the mixture was purified by ether precipitation to completely remove the impurity. Then the creamy yellow reaction product was obtained by vacuum drying.

Synthesis of acid-sensitive programmed Tf-targeted PCL-DAK-PEG-T7 (PDPT): The D-T7 peptide (1 mg) and PDP (12 mg) were dissolved into 3 mL acetone, and then TEA (15 μL) were added to maintain the alkaline environment. The next operation was the same as that of PPM.

Preparation of Asp@TMNPs: To give an overall comparison, PP, PPM, PDP, PDPT and methoxyl PCL-PEG (PCL-MPEG) were prepared. A total of 2.4 mg aspirin, 4.6 mg PCL-MPEG, 0.55 mg PPM and 1.625 mg PDPT were dissolved in 100 μL acetone and then mixed with 2 mL deionized water. With the ultrasonic emulsification (100 W, 5 s/ 5 s, 5 min), the Asp@TMNPs was formed.

Characterization: The particle size and zeta potential of the complexes were evaluated using the dynamic light scattering technique on a Malvern meter (Malvern, UK). The morphology was observed using a transmission electron microscope (TEM, H-600, Hitachi, Japan). The drug-loading capacity and encapsulation efficiency of aspirin were measured by

high performance liquid chromatography (Agilent LC-20A, USA), which were referred to the standard of “Chinese Pharmacopoeia” (ch. P, 2015 edition).

Serum stability: The plasma stability of different formulations was evaluated in PBS with different concentration of fetal bovine serum (FBS). Asp@TMNPs and controlled nanoparticles were suspended in PBS with 0%, 10% and 50% FBS and incubated under the condition of 75 rpm, 37°C. The particle size was determined by dynamic light scattering.

Cytotoxicity assay: Cytotoxicity was assessed using an MTT assay. Briefly, bEnd.3 cells were grown on 96-well plates by the concentration of 5×10^3 cells per well. Then different concentrations of drugs, empty nanoparticles and drug-loaded nanoparticles were incubated with the cells for 12 h. Subsequently, the MTT assay was used to determine cell viability. Untreated cells were used as the negative controls, and the viability was expressed as the percentage of the absorbance of the negative control.

Preparation of Cou6@TMNPs: A total of 1.5 μL Coumarin-6 (1 μg mL^{-1}), 4.6 mg PCL-MPEG, 0.55 mg PPM and 1.625 mg PDPT were dissolved in 100 μL acetone and then mixed with 2 mL deionized water. With the ultrasonic emulsification, the Cou6@TMNPs was formed.

Preparation of Did@TMNPs: A total of 1.0 mg DiD, 67.9 mg PCL-MPEG, 8.1 mg PPM and 24 mg PDPT were dissolved in 1.5 mL acetone and then mixed with 30 mL deionized water. With the ultrasonic emulsification, the Did@TMNPs was formed.

Antibodies Western Blotting: Cells were lysed using radioimmunoprecipitation assay (RIPA) buffer (P0013C, Beyotime) containing a protease and phosphatase inhibitor cocktail (B14011 and B15001, respectively, Bimake) to extract the total protein. Equal amounts of denatured protein samples were separated on 10% SDS-polyacrylamide gels and transferred to a polyvinylidene difluoride (PVDF) membrane (IPVH00010, Millipore) for immunoblot analysis. GAPDH was used as an internal control, and the intensity of bands was analyzed using Image J software (version 1.5).

Quantitative PCR: BV2 cells were cultured and treated as described earlier, and total RNA was extracted using TRIzol reagent (Invitrogen, 15596026). A NanoDrop 2000 (Thermo Scientific) was used to measure the amount of total RNA in each sample, and 500 ng of total RNA was subjected to reverse transcription (RT) using an RT reagent kit (Takara, RR037A) to form cDNA. Quantitative PCR for cDNA was performed in triplicate using SYBR Green qPCR Master Mix (Bimake, B21202) on an iCycler RT-PCR detection system (Bio-Rad Laboratories). GAPDH was used as an internal control. Delta-delta Ct value analysis was used to evaluate the relative expression of target genes. Primer pairs were designed using Primer3plus (http://www.bioinformatics.nl/cgibin/ primer3plus/primer3plus.cgi).

Staining and immunofluorescence: For MitoSox (M36008, Invitrogen) staining, BV2 cells were cultured on glass coverslips and washed with 1× Hank's balanced salt solution (HBSS) (14065056, Gibco). MitoSox was loaded onto the cells and incubated for 15 min at 37°C, protected from light. Then cells were washed three times with warm 1× HBSS and counterstained with 4,6-diamidino-2-phenylindole (DAPI, P0131, Beyotime) to label the nuclei. At least five quality images were acquired using a laser scanning confocal microscope (Olympus FV2000).

For immunofluorescence, BV2 cells were cultured on glass coverslips, washed with Dulbecco's phosphate-buffered saline (DPBS) (14190144, Gibco), fixed with 4% paraformaldehyde, permeabilized with 0.3% Triton X-100 (T8200, Solarbio), blocked with 5% bovine serum albumin (BSA, 4240GR500, Biofroxx) in PBS, and incubated with primary antibodies overnight at 4°C. Then, the slides were washed in PBS, incubated with Alexa Fluor 594-labeled secondary antibodies for 1 h at room temperature, and counterstained with DAPI to label the nuclei. At least five quality images were acquired using a laser scanning confocal microscope (Olympus FV2000).

MIA Induced Autism-like Animal Model and Intervention: C57BL/6 male mice (n = 10, 7~8 weeks, 22~25 g) for each group and were housed in 24 ± 1℃. Male and female mice were co-

caged with 1:1, and then we count the day after mating with virginal plug (G0.5). Pregnant mice were divided with control group and MIA treatment group. During the pregnant mice for 12.5 day, MIA mice were injected (polyinosinic polycytidylic acid, PolyI/C) 20 mg kg^{-1}, control group was treated with PBS. After postnatal for 21 days (PND21), the male and female mice were housed separately. Then after (PND21), MIA groups were randomly divided into PolyI/C and Asp@TMNPs group, with the dose of aspirin (40 mg kg^{-1}), were treated after another day for 21 days.

2. Supporting results

Table S1. Characterization of nanoparticles. Data were presented as mean ± SD (n = 3).

	Number (nm)	ξ-potential (mV)	PDI	Drug-loading Capacity (%)	Encapsulation Efficiency (%)
Asp@NPs	62.3±1.9	9.92±0.99	0.101±0.015	23.6±0.4	88.4±1.3
Asp@MNPs	59.7±0.6	6.28±2.95	0.141±0.008	23.9±0.5	89.0±1.4
Asp@TNPs	63.4±1.6	8.36±2.91	0.111±0.009	24.2±1.6	92.0±6.1
Asp@TMNPs	62.2±3.1	8.92±1.36	0.129±0.001	19.7±2.3	75.2±8.6

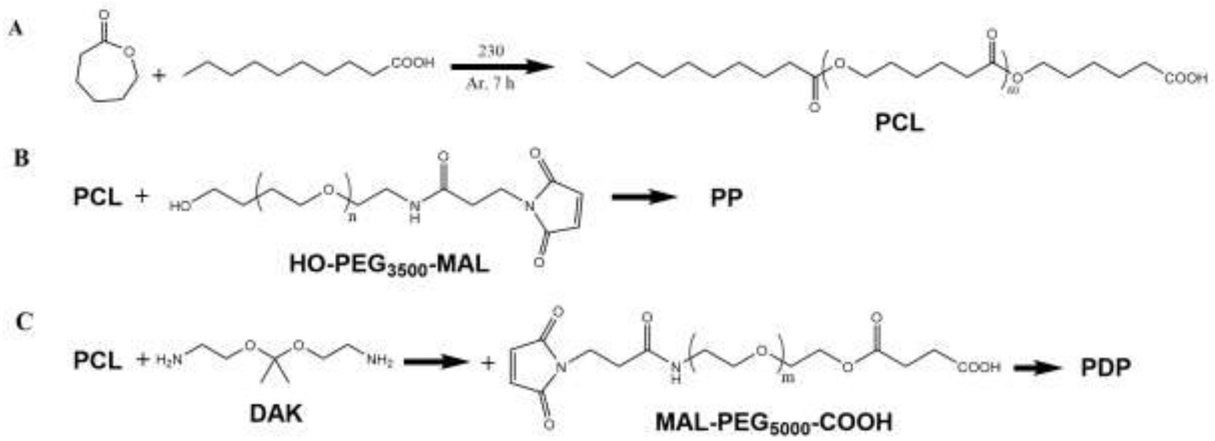

Figure S1. Synthetic routes of intermediate products: (A) PCL, (B) PP, (C) PDP.

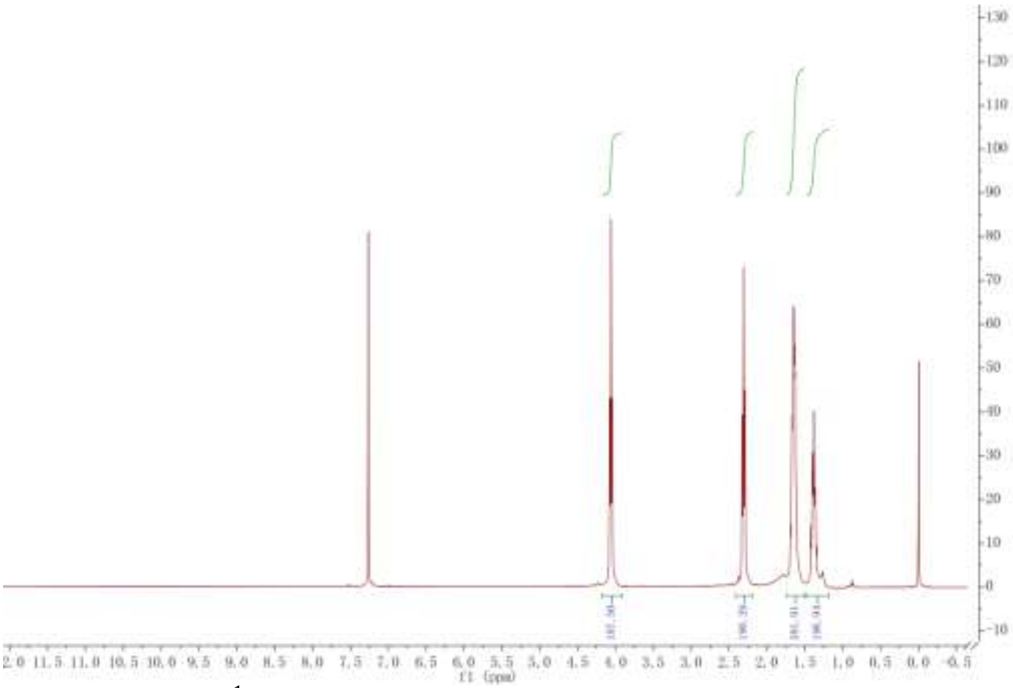

Figure S2. NMR ^{1}H spectrum of PCL.

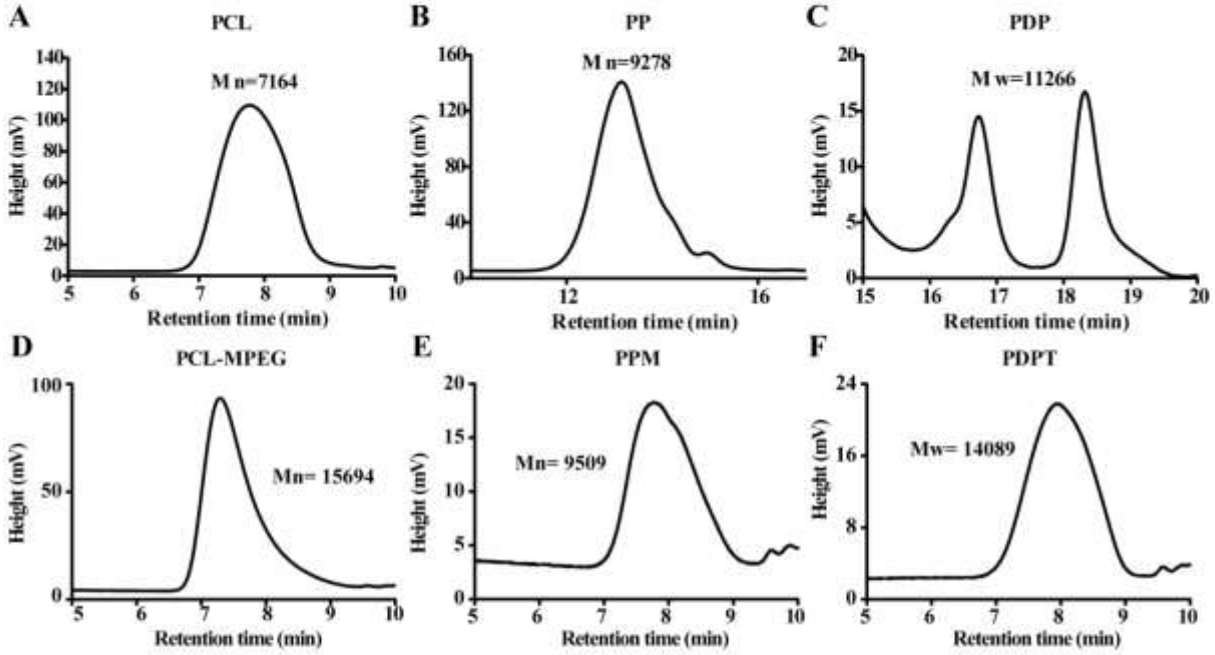

Figure S3. GPC of intermediate product: (A) PCL, (B) PP, (C) PDP, (D) PCL-MPEG, (E) PPM, (F) PDPT.

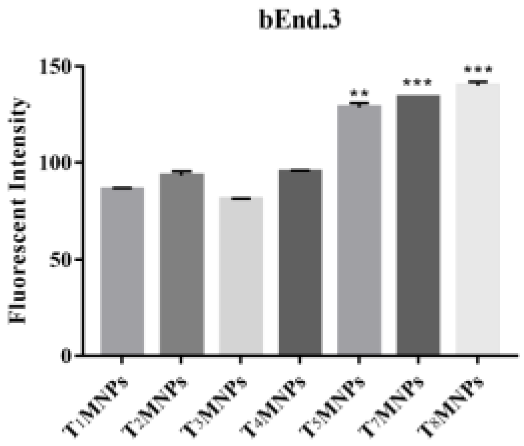

Figure S4. Uptake of bEnd.3 cells after treating with different concentration of D-T7 modified nanoparticles for 4 h. Data were presented as mean ± SD (n = 3) and the p values were comparative analysis with T_1MNPs. P>0.05, 0.01≤P<0.05, 0.001≤P<0.01 and P<0.001 were remarked with ns, *, **, and ***, respectively.

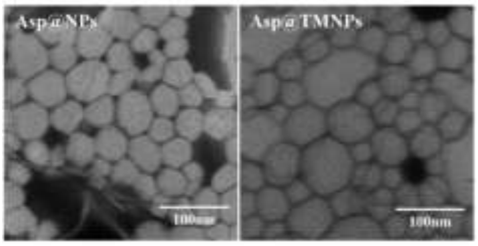

Figure S5. TEM images of Asp@NPs and Asp@TMNPs.

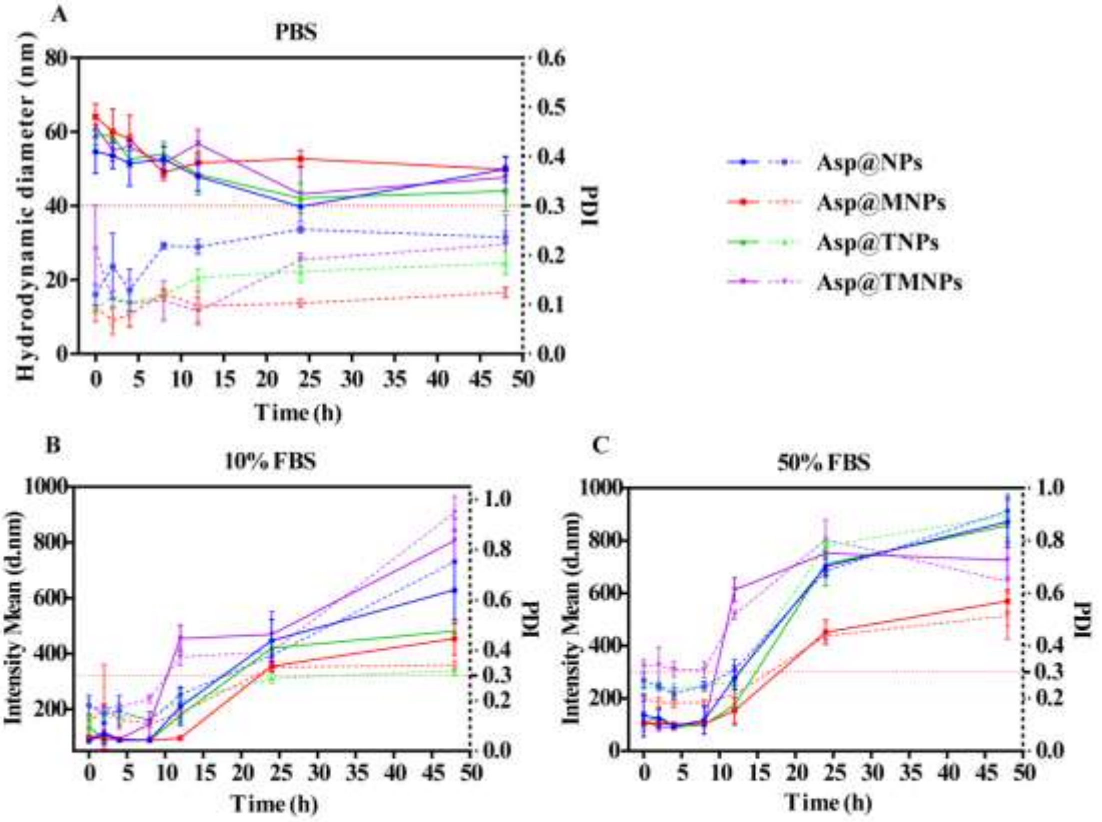

Figure S6. Stability of Asp@TMNPs and controlled nanoparticles in different concentrations of FBS. (A) 0%FBS, (B) 10% FBS, (C) 50% FBS. Data were presented as mean ± SD (n = 3).

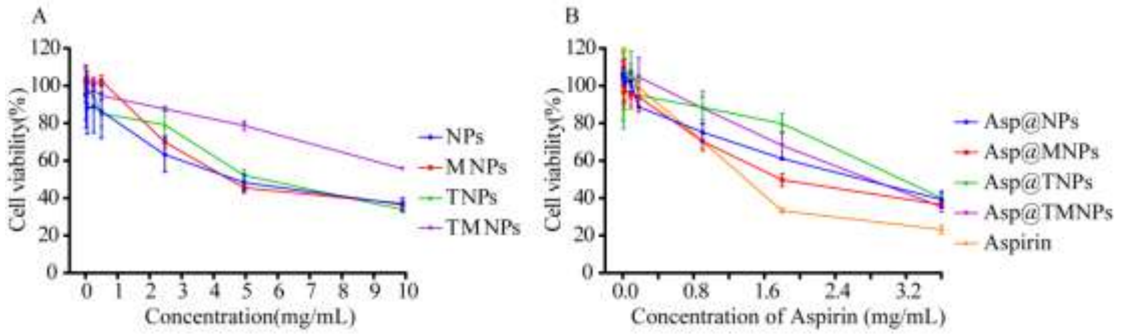

Figure S7. The bEnd.3 cell viability incubated with different formulations measured by MTT assay. (A) Empty nanoparticles. (B) Aspirin and aspirin formulations. Data were presented as mean ± SD (n = 3).

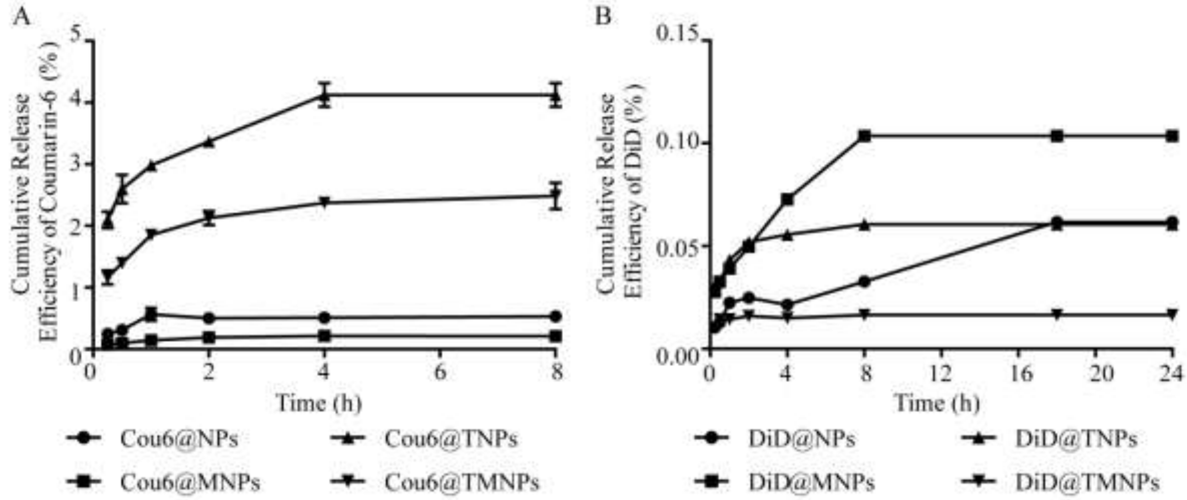

Figure S8. Cumulative release efficiency of Coumarin-6 and DiD loaded nanoparticles incubated in PBS buffer. Data were presented as mean ± SD (n = 3).

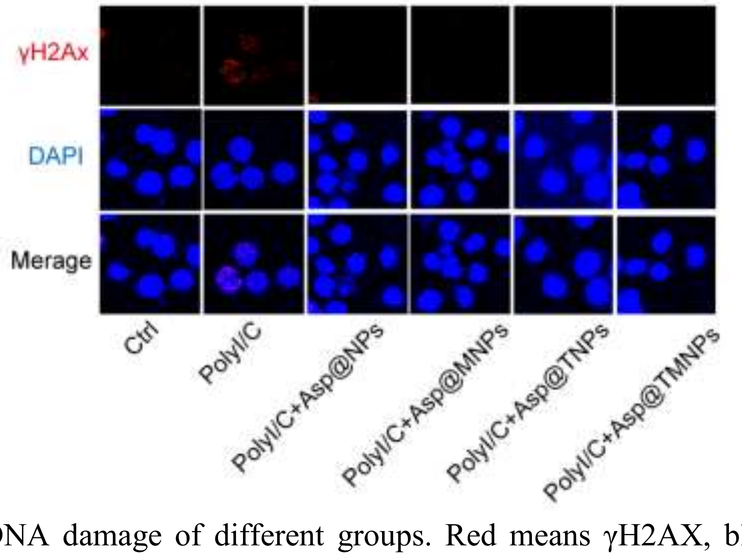

Figure S9. The DNA damage of different groups. Red means γH2AX, blue means DAPI. PolyI/C transfection induced BV2 cell DNA damage and increased inflammation markers, which could be rescued by asprin.

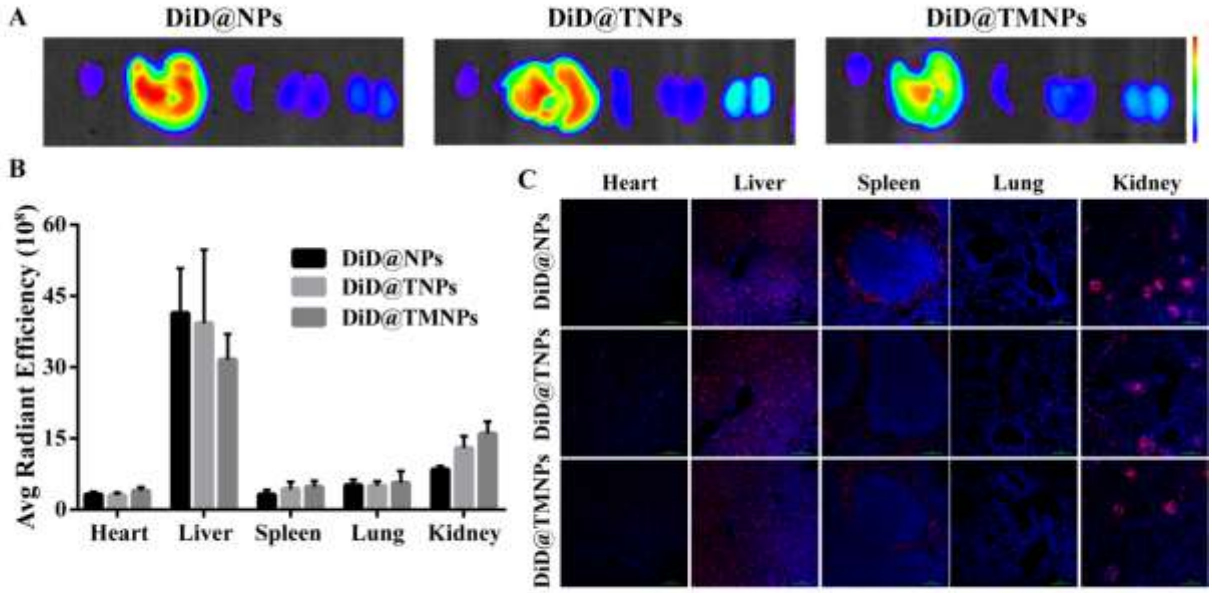

Figure S10. In vivo distribution. (A) Ex vivo imaging of organs in different groups after 12 h. (B) The semiquantitative fluorescence intensity of organs. Data were presented as mean ± SD (n = 3). (C) Representative confocal fluorescence images of organs showing the accumulation of different nanoparticles. Scale bar represents 50 µm.
